# Facial mimicry is not modulated by dopamine D2/3 and opioid receptor antagonism

**DOI:** 10.1007/s00213-023-06426-3

**Published:** 2023-07-21

**Authors:** Sebastian Korb, Alasdair Clarke, Claudia Massaccesi, Matthäus Willeit, Giorgia Silani

**Affiliations:** 1 Department of Psychology, University of Essex, Essex, UK; 2 Department of Cognition, Emotion, and Methods in Psychology, University of Vienna, Vienna, Austria; 3 Department of Psychiatry and Psychotherapy, Medical University of Vienna, Vienna, Austria; 4 Department of Clinical and Health Psychology, University of Vienna, Vienna, Austria

**Keywords:** Facial mimicry, Dopamine, Opioid, Amisulpride, Naltrexone, Embodiment, Electromyography

## Abstract

**Rationale:**

According to theories of embodied cognition, facial mimicry — the spontaneous, low-intensity imitation of a perceived emotional facial expression — is first an automatic *motor* response, whose accompanying proprioceptive feedback contributes to emotion recognition. Alternative theoretical accounts, however, view facial mimicry as an emotional response to a *rewarding* stimulus, and/or an affiliative signal, and thus reject the view of an automatic motor copy.

**Objectives:**

To contribute to this debate and further investigate the neural basis of facial mimicry, as well as its relation to reward processing, we measured facial reactions to dynamic happy and angry faces after pharmacologically manipulating the opioid and dopamine systems — respectively, thought to subserve ‘liking’ and ‘wanting’ of rewards.

**Methods:**

In a placebo-controlled, double-blind experiment, 130 volunteers received in a between-subjects design 50 mg of the opioidergic antagonist naltrexone, 400 mg of the dopaminergic antagonist amisulpride, or placebo.

**Results:**

Clear occurrence of facial mimicry, measured 4 h after drug intake with electromyography (EMG) of the zygomaticus major and corrugator supercilii muscles, was found. However, facial mimicry was not affected by either compound, as shown with both frequentist statistics, and a Bayesian asymptotic regression model.

**Conclusions:**

This null finding does not support the hypothesis that facial mimicry (of happiness) reflects an emotional response to a rewarding stimulus, leaving open the possibility of facial mimicry being an automatic motor copy. The results are relevant to the discussion about the psychological nature and the neural basis of facial mimicry, although they should be considered preliminary, given the challenges of interpreting null findings when targeting a novel effect of unknown size.

**Supplementary Information:**

The online version contains supplementary material available at 10.1007/s00213-023-06426-3.

## Introduction

The human face is a rich means of nonverbal communication and can constitute a powerful social reward (Chelnokova et al. 2014), especially when displaying positive emotions, such as smiles (Izuma 2015). Observing others’ emotional facial expressions also elicits facial mimicry, i.e. the partial, low-intensity imitation of the expression in the face of the perceiver (Dimberg 1982). Several theoretical accounts have been proposed to explain the origin and effect of facial mimicry.

A heated debate persists around whether facial mimicry contributes to emotion recognition. Theories of embodied cognition (Niedenthal et al. 2005), which have their origins in the nineteenth-century work by Darwin, James, and Lange (Darwin 1872; James 1884), and later generated the facial feedback hypothesis (Coles et al. 2019; Davis et al. 2009), propose that emotional facial expressions are automatically copied and that the ensuing congruent changes in the observer’s own facial muscle contractions are fed back to their somatosensory cortices and other brain areas, where they influence the recognition (and possibly the visual perception) of the visual stimulus (Wood et al. 2016b). This claim that facial feedback contributes to emotion recognition (also called the ‘matched motor’ hypothesis of mimicry) has received considerable support from empirical studies (e.g. Marmolejo-Ramos et al. 2020; Niedenthal et al. 2001; Rychlowska et al. 2014; Stel and van Knippenberg 2008; Wood et al. 2016a), but has recently come under attack, largely due to the failed replication of certain prominent findings (Wagenmakers et al. 2016). A meta-analysis, and a recent multi-laboratory pre-registered collaboration involving nearly 4000 participants, has however confirmed that facial feedback effects exist and can initiate and amplify feelings of happiness (Coles et al. 2022a, b, 2019). Important individual and situational differences exist, however, in the propensity to engage in facial mimicry. For example, smile mimicry is reduced for faces associated with losing compared to winning money, and joint facial electromyography (EMG) and functional magnetic resonance imaging (fMRI) recordings suggest this could be due to top-down inhibition from the medial prefrontal cortex (Hofman et al. 2012; Korb et al. 2019; Sims et al. 2012).

In contrast, the ‘contextual’ or ‘mimicry as social regulator’ view proposes that facial mimicry results from an affiliative intention and reflects emotion understanding (Hess and Fischer 2022, 2014). In this perspective, facial mimicry is the product of, rather than a contributing source to, the recognition of other people’s emotional expressions. The emphasis is on how emotions are mimicked more in certain situations, e.g. when expressed by in-group than out-group members (Seibt et al. 2015). Importantly, if facial mimicry is the expression of an affiliative intent following the recognition of an emotion in context, then mimicry of smiles, which are considered rewarding stimuli, should also be modulated by drugs affecting the chemical basis of the brain’s reward system.

Rewards are powerful emotion elicitors and share many commonalities with emotions (Sander and Nummenmaa 2021). Broadly speaking, the neuroscience of reward distinguishes between motivational and hedonic responses, i.e. ‘wanting’ and ‘liking’, respectively, believed to be grounded in the dopaminergic and opioidergic systems (Berridge and Kringelbach 2015). Based on the (mostly separated) literatures on the processing of facial expressions as emotional stimuli or rewards, facial mimicry of smiles can therefore be understood as an emotional response to a rewarding stimulus (the other’s smiling face) which is driven by an affiliative motivation (‘wanting’) and/or by the pleasure elicited by its encounter (‘liking’).

The manipulation of the major underlying neurochemical brain systems regulating reward processing (i.e. dopamine and opiates) constitutes a promising tool to further explore the nature of facial mimicry. Pharmacologically challenging either of these neurochemical systems should indeed impact facial mimicry, and an eventual asymmetry in the effects of dopaminergic and opioidergic manipulations would reveal if facial mimicry reflects more wanting or liking — although some changes in affiliative motivation would also be expected following opioid modulation, given that the opioid system is thought to be, together with oxytocin, the main system behind affiliations. On the other hand, if changes to the dopamine and/or opioid systems have no effects on facial mimicry, then the hypothesis that facial mimicry is primarily an anticipation of, or a response to, a reward is not supported, and instead the hypothesis that facial mimicry is the result of an automatic motor copy becomes more likely. The goal of the experiment reported here was to test these two contrasting hypotheses.

Interest in the modulation of facial mimicry by changes in the brain’s levels of hormones and neurotransmitters, such as testosterone, oxytocin, vasopressin, and opioids, has recently increased (Hermans et al. 2006; Korb et al. 2016a; Meier et al. 2016; for a review see Kraaijenvanger et al. 2017), but preliminary findings about the role of the opioid system are inconsistent. For example, reduced frowning, but no change in smiling, have been observed in response to happy faces after administration of 50 mg of naltrexone, an opioid receptor antagonist (Meier et al. 2016), while another study found no significant effects of 25 or 50 mg of naltrexone on the facial mimicry of angry, fearful, happy, and sad facial expressions (Wardle et al. 2016). Recently, we reported some reduction in the mimicry of fear, but no effects on mimicry of happiness or anger, after administration of 10 mg of morphine, a highly selective mu-opioid receptor agonist (Massaccesi et al. 2022). Even less is known about the role of the dopamine system in facial mimicry. Evidence suggests that people with Parkinson’s disease — who have lower dopamine levels in the basal ganglia and other brain areas (Poewe et al. 2017) — have reduced facial mimicry, as well as impaired emotion recognition, especially for happiness (Argaud et al. 2016). However, pharmacological challenges in healthy participants, which could corroborate the role of dopamine for facial mimicry, are scarce. One notable example is the finding by Wardle and de Wit (2014) of greater corrugator relaxation in response to happy faces after administration of MDMA, which primarily affects the serotonin and noradrenaline system, but also interacts with dopamine and several other neurotransmitter systems.

In summary, the currently available evidence is scarce and mixed, but suggests that opioids, and potentially also dopamine, may play a role in the facial mimicry response by acting on the motivation to affiliate and/or the hedonic pleasure related to it. However, to date, a direct comparison of the effects of dopaminergic and opioidergic modulations on facial mimicry is lacking. In order to fill this gap, the following experiment investigated the roles of the dopamine and opioid systems in facial mimicry by studying healthy participants in a double-blind placebo-controlled pharmacological intervention. Using a between-subjects design, participants received 400 mg of amisulpride (a dopamine D2/3 receptor antagonist), 50 mg of naltrexone (an opioid receptor antagonist), or a placebo. Four hours after drug/placebo administration, facial mimicry of dynamic happy and angry facial expressions morphing into each other was measured with facial EMG of the corrugator supercilii (CS) and zygomaticus major (ZM) muscles.

Based on the understanding that pleasure responses are promoted/inhibited by opioid agonists/antagonists (Nummenmaa and Tuominen 2018), and given the assumption that smiling faces constitute rewards, while angry faces are perceived as non-rewarding or as punishments, we specifically expected drug effects on facial mimicry of smiles, i.e. the activation of smiling muscles (ZM) and relaxation of frowning muscles (CS). Furthermore, we expected reduced smile mimicry in the naltrexone compared to the placebo group, suggesting that mimicry reflects pleasure derived by reward consumption, or an affiliative motivation (Chelnokova et al. 2014; Meier et al. 2016), and/or in the amisulpride group compared to placebo, suggesting that facial mimicry may constitute a motivation to affiliate, driven by anticipatory pleasure. In contrast, if facial mimicry initially/primarily reflects an automatic motor copy, as suggested by theories of embodied cognition, then it should not be modulated by either compound (as long as dopamine transmission is not blocked to the point to interfere with general motor activity).

## Methods

### Participants

Based on previous work that had used the same compounds and doses (Weber et al. 2016), we aimed to collect data from 40 participants per group. The final sample included 130 volunteers (87 females) aged 18–35 years (*M* = 23.2; *SD* = 3.55) and sample sizes per group ranged 42–44 (see Table 1). All participants reported being right-handed, to smoke less than five cigarettes daily, to have no history of current or former substance use, and to be free of psychiatric or neurological disorders. Participants’ average body mass index (BMI) was 22.6 (*SD* = 2.49, range 17.7–29.3). The study was performed in line with local ethics regulations and the Declaration of Helsinki (World Medical Association 2013). Participants signed informed consent and received monetary compensation of 10 € per hour.

**Table 1:** Demographic and individual differences by drug group

	Amisulpride	Naltrexone	Placebo	Total/Mean	*p*
N	42	44	44	130	0.9
N females	28	30	29	87	0.9
N males	14	14	15	43	0.9
Age	23.7 (4.2)	22.9 (2.8)	22.9 (3.6)	23.2	0.3
BMI	22.67 (2.56)	23.01 (2.31)	22.15 (2.60)	22.6	0.3
Hours from pill	4.2 (0.2)	4.2 (0.3)	4.1 (0.2)	4.2	0.6
PANAS + (t1–t2)	3.5 (4.8)	5.1 (4.9)	2.7 (3.7)	3.7	0.06
PANAS—(t1–t2)	1.2 (1.5)	2.3 (5.5)	1.0 (1.8)	1.44	0.2
N guess PLA	24	18	22	64	0.6
% corr guess	16.7	29.5	50	32.3	< 0.001
Nausea (t1)	1.05 (0.2)	1.02 (0.1)	1.00 (0.0)	1.02	0.14
Nausea (t2)	1.00 (0.0)	1.20 (0.6)	1.00 (0.0)	1.07	0.93

*PANAS*, Positive ( +) and Negative (-) Affect Schedule; *t1*, immediately before pill intake; *t2*, 3 h after pill intake; t1–t2 indicates the difference score; *PLA*, placebo.

### Stimuli and task

The stimuli and task have been described in previous publications (Korb et al. 2016a, b). A total of 24 videos (duration 5 s at 25 frames per sec) were created, based on photos of 10 faces (five male) with happy and angry expression, using morphing software (Morpheus Photo Morpher, version 3.17). Each video displayed a happy face gradually becoming angry, or vice versa and was repeated four times, for a total of 96 trials shown in semi-random order, with a maximum of three successive stimuli with the same emotion, in two blocks of 48 trials.

Participants were instructed to indicate for each video the moment at which the first expression changes into the second, by pressing with their left middle finger the arrow-up button on a small number keypad. In each trial, a fixation cross was shown at the centre of the screen for 2–3 s (average duration 2.5 s), immediately followed by the video (5 s), and a feedback screen for 1 s. The feedback screen was blank in correct trials, while it contained the text “Do not forget to press” if no button had been pressed during the video, the text “Wrong button (only arrow up)” if the wrong button had been pressed, or the text “Please press only once” if the correct button had been pressed more than once. The task was preceded by four practice trials.

### EMG

After cleansing of the corresponding face areas with alcohol, water, and an abrasive paste, Ag/AgCl electrodes were attached bipolarly according to guidelines on the left corrugator supercilii (CS) and the zygomaticus major (ZM) muscles (Fridlund and Cacioppo 1986). A ground electrode was attached to the participants’ forehead and a reference electrode on the left mastoid. EMG data were sampled at 1200 Hz with impedances below 20 kOHM using a g.USBamp amplifier (g.tec Medical Engineering GmbH) and the MATLAB software (The MathWorks, Inc.).

### Procedure

The here reported facial mimicry experiment was carried out as part of a series of other tasks investigating the processing of food and touch rewards (Korb et al. 2020a), as well as risk-taking and working memory (Mikus et al. 2022). Participants’ physical (blood draw, ECG) and mental (semi structured psychiatric interview) health were checked on a first laboratory visit. On their second laboratory visit (3–60 days later), following a negative drug and urine pregnancy test, participants received in a between-subjects design a pill administered by the study doctor containing either 400 mg of amisulpride, 50 mg of naltrexone, or mannitol (placebo). The facial mimicry task started about 4.2 h after drug intake (Table 1, Figure S1). This delay is appropriate given the pharmacodynamics of the two drugs. Serum levels of Amisulpride peak after approximately 4 h and have an elimination half-life of 12 h (Rosenzweig et al. 2002). Naltrexone reaches maximal concentration in plasma after 1 h, but with a 50 mg dose over 90% of mu-opioid receptors remain blocked for at least 49 h (Trøstheim et al. 2023).

To test if groups differed by mood, participants filled out the Positive and Negative Affect Schedule (PANAS Watson et al. 1988) at the beginning of the second laboratory session (immediately before pill intake) and again 3 h after pill intake. During testing, participants were seated at a table, at an approximate distance of 60 cm from an LCD monitor with a resolution of 2560 × 1600 pixels. The task was run on a desktop computer with Windows XP using MATLAB 2014b, The MathWorks, Natick, 2014 and the Cogent 2000 and Cogent Graphics toolboxes.

### Analyses

Data and analysis scripts are available in the OSF repository (https://osf.io/uht37). Data were statistically analysed in R (R Core Team 2020).

#### Response times

Response times (RT) were converted to z-scores using the function *scale_within* from the package *mousetrap*. A series of chi-square goodness of fit tests and one-way ANOVAs were used to investigate demographic and other basic differences between groups (Table 1). For each subject, trials with an RT greater/smaller than the subject’s mean + / − 2 times the standard deviation were marked as outliers and excluded from analyses. In total, 2.44% of all trials were rejected as behavioural outliers, and the number of rejected trials did not differ by drug group (*F*(1,128) = 0.01, *p* = 0.89). We then conducted frequentist statistics on RT data by fitting a linear mixed-effects model (LMM) including the fixed effects emotion (HappyToAngry, AngryToHappy) and drug (amisulpride, naltrexone, placebo), and as random effects by-subject and by-stimulus intercepts and slopes by emotion.

#### Facial EMG

EMG data were pre-processed in MATLAB, partly using the EEGLAB toolbox (Delorme and Makeig 2004). A 20 to 400 Hz bandpass filter, as well as a 50 Hz notch filer, was applied, and then data were rectified and smoothed with a 40 Hz low-pass filter. Epochs were extracted from 0.5 s before to 5 s after stimulus onset and expressed as percentage of baseline (the average of the 500 ms preceding stimulus onset). Trials with average values more than 2 SDs above or below the mean (for that subject and muscle), and with peak values more than 2 SDs from the average peak, were removed from analyses. Applying the same procedure, trials were also removed if outlier values were detected in the baseline period. This resulted in the overall exclusion of 19.6% of all trials. The number of EMG outliers did not differ between groups (*F*(1,128) = 0.35, *p* = 0.56). Data were then converted to proportion of baseline (e.g. a value of 0 indicates 0% of the baseline, and a value of 1 indicates 100% of the baseline) and log transformed, to account for their skewness.

To investigate if facial mimicry occurred and was modulated by the drugs, we conducted frequentist statistics on the log EMG data by fitting a separate LMM per muscle with the fixed effects emotion (HappyToAngry, AngryToHappy), drug (amisulpride, naltrexone, placebo), and time (5 windows of 1 s), and with random intercepts by subject and stimulus-face, and with by-subject random slopes for emotion, time, and their interaction. These frequentist LMMs were followed up by Bayesian statistics that also modelled an asymptotic regression to account for the non-linear muscle activation/relaxation changes over time in each trial.

## Results

### Behaviour

The three drug groups (see Table 1) did not differ in number of participants overall (× 2(2) = 0.06, *p* = 0.9), number of female participants (× 2(2) = 0.07, *p* = 0.9), number of male participants (× 2(2) = 0.05, *p* = 0.9), age (*F*(1,127) = 0.62, *p* = 0.5), BMI (*F*(1,127) = 1.33, *p* = 0.3), task time since pill intake (*F*(1,127) = 0.15, *p* = 0.9), and pre-post difference in positive mood (*F*(1,122) = 2.97, *p* = 0.06, or negative mood (*F*(1,123) = 1.59, *p* = 0.2). The percentage of participants correctly guessing their condition was significantly different across the groups (× 2(2) = 17.6, *p* < 0.001). This was due to most participants believing they were part of the placebo condition, which naturally resulted in a higher percentage of correct guesses in the actual placebo group. Crucially, participants in the two drug groups were blind to their condition, as their percentages of correct guessing (16.7% and 29.6%) were both below chance level (33.3%). The three groups of participants also did not differ in terms of self-reported nausea (a common side effect of naltrexone) at time of pill intake and three hours later (see Table 1).

LMM analysis on RTs did not reveal significant main or interaction effects (all *F*s < 1.4, all *p*s > 0.26), suggesting that the performance was neither modulated by the emotion nor the drug group (Figure S2).

### Facial EMG

#### Frequentist statistics

**Fig. 1:**
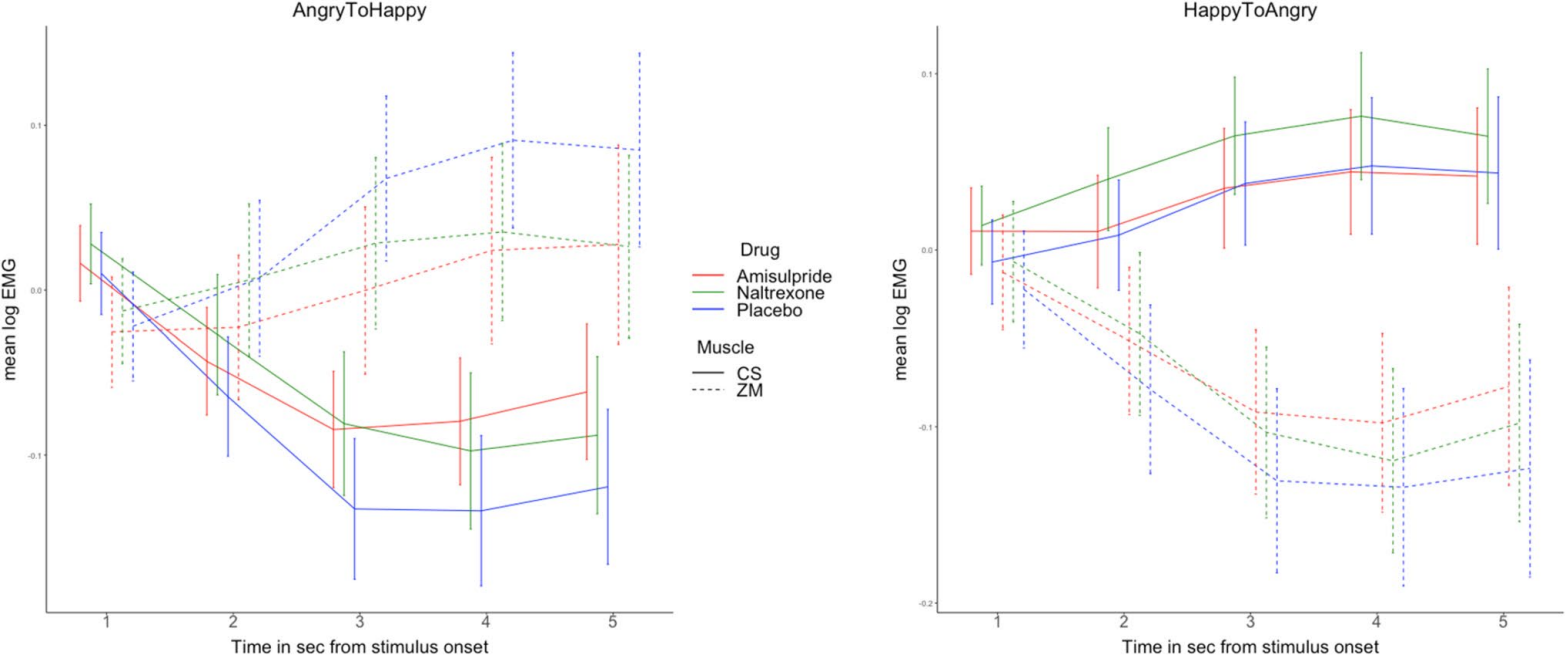
Facial EMG by muscle, time, and drug. During AngryToHappy trials (left), facial mimicry of smiles, as shown by ZM activation and CS deactivation, was somewhat greater in the placebo condition, but no significant differences between drug groups were found. Similarly, during HappyToAngry stimuli, facial mimicry of anger was present, as shown by CS activation and ZM deactivation, but was not modulated by either drug. Error bars represent SEM. CS, corrugator supercilii muscle; ZM, zygomaticus major muscle

The LMM on the ZM muscle resulted in a statistically significant main effect of emotion (*F*(1, 126.64) = 51.2184, *p* < 0.001), and an emotion × time interaction (*F*(1, 125.23) = 61.4159, *p* < 0.001). The interaction reflected facial mimicry of happiness, i.e. increasing ZM activation across the five time windows of each trial for AngryToHappy trials (*b* = 0.0187, SE = 0.00416, z = 4.488, *p* < 0.001) and decreasing ZM activation across time for HappyToAngry (*b* =  − 0.0226, SE = 0.00254, *z* =  − 8.901, *p* < 0.001). A marginally significant emotion × drug interaction (*F*(2, 126.64) = 2.5438, *p* = 0.08) pointed to a more pronounced ZM activation for AngryToHappy trials (Fig. 1, left), and ZM deactivation for HappyToAngry trials (Fig. 1, right), in the placebo condition, especially compared to the amisulpride group. We followed up on this marginal effect by fitting a smaller model on the ZM muscle in AngryToHappy trials only (dropping the factor emotion). This resulted only in a significant main effect of time (*F*(1, 126.75) = 20.0643, *p* < 0.001), but both the main effect of drug and the drug × time interaction were not significant (both *F* < 2, *p* > 0.14). In summary, although facial mimicry of happiness was clearly present (in terms of both ZM contraction to happiness and ZM relaxation to anger), it was not significantly modulated by neither of the two drugs.

To investigate if facial mimicry of anger occurred in the CS muscle, and whether it differed between drug groups, a similar LMM was fitted with the log of the EMG of the CS as dependent variable. This resulted in statistically significant main effects of emotion (*F*(1, 126.67) = 94.6301, *p* < 0.001), and time (*F*(1, 126.76) = 18.0572, *p* < 0.001), as well as an emotion × time interaction (*F*(1, 125.91) = 93.2686, *p* < 0.001). The interaction indicated facial mimicry of anger, i.e. increasing CS activation across the five time windows of HappyToAngry trials (*b* = 0.0123, SE = 0.00231, *z* = 5.347, *p* < 0.001, see Fig. 1, right), and decreasing CS activation across time for AngryToHappy trials (*b* =  − 0.0275, SE = 0.00308, *z* =  − 8.910, *p* < 0.001, see Fig. 1, left). Both the main effect of drug and its interaction with time and emotion were not significant ( *F*s < 2, *p*s > 0.1). In summary, facial mimicry of anger was clearly present (CS contraction to anger and CS relaxation to happiness), but it was not modulated by the drug group.

#### Bayesian statistics

We further explored the data using a non-linear (asymptotic) Bayesian model, which also attempted to achieve an improved nonlinear fit. As seen from the frequentist statistics above, EMG signals approach an asymptote (i.e. ceiling and floor effects). This may have negatively affected our ability to detect significant drug effects (see above). Hence, we accounted for this data pattern using asymptotic regression and fitting the curve described by the equation below:

$$y= a - \left(a-b\right)* {e}^{(-exp(c) * t-1)}$$

where *t* is time and *a*, *b*, and *c* are fitted to the data. The asymptote and rate are represented by *a* and *c*, respectively. We allowed both to vary over our 2 × 2 conditions and included a maximal random effects structure. The rate of change was modelled to always be positive (from the starting position *b* to the asymptote). We used a log link, hence the *exp(c)* term in the equation above. Finally, *b* represents the starting value at *t* = 1, which we assumed has a fixed effect of 0 (given that data was expressed as percentage of the baseline), but has a maximal random effects structure. More explicitly:

$$a \sim 0 + drug:muscle:emotion + (0 + muscle:emotion|sub)$$

$$b \sim 0 + (0 + muscle:emotion|sub)$$

$$c \sim 0 + drug: muscle:emotion + (0 + muscle:emotion|sub)$$

**Fig. 2:**
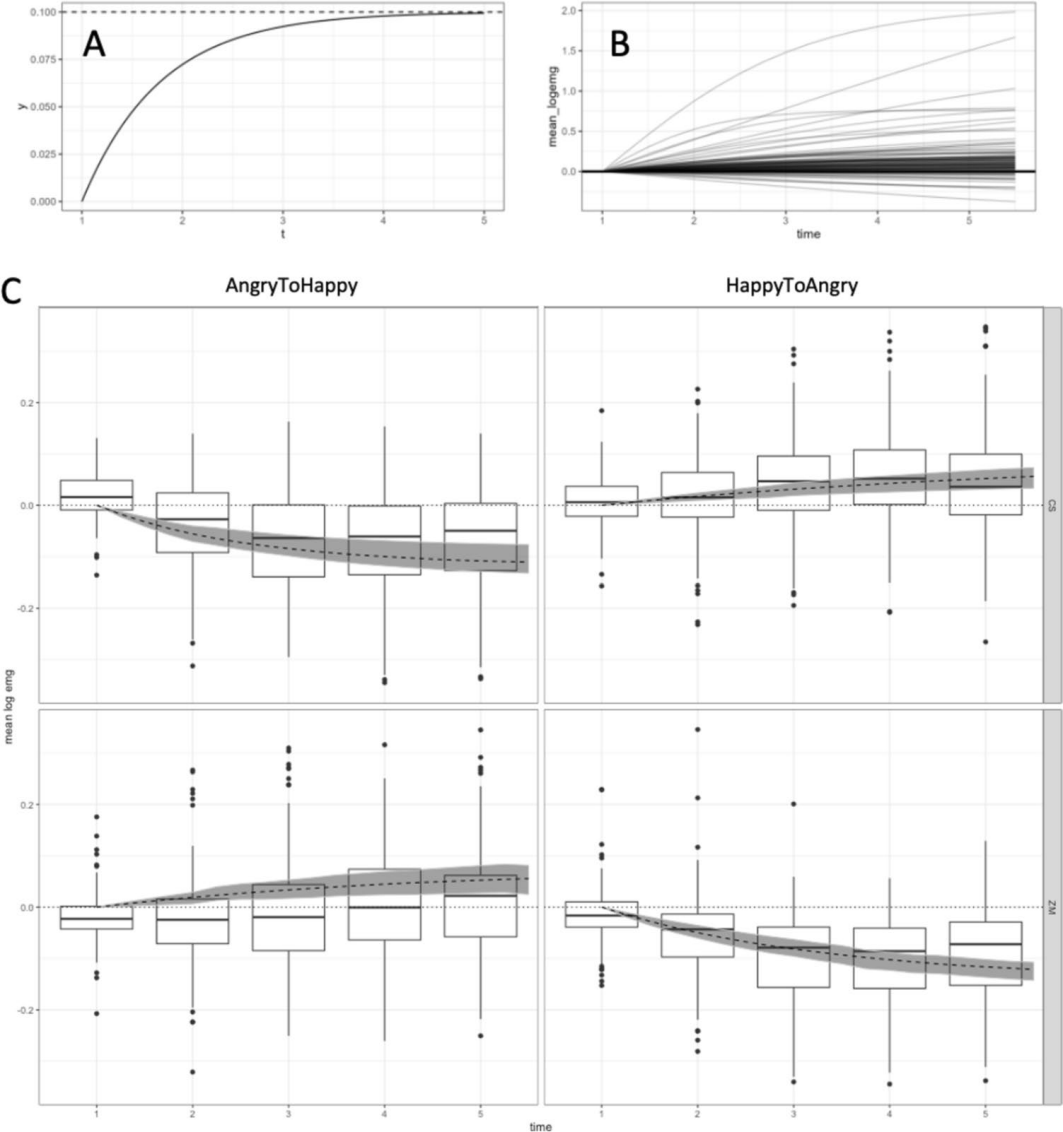
**A** An asymptotic regression line with a = 0.1, b = 0 and c = 0.25. The dashed line indicates the asymptote. **B** A prior prediction plot. Each line shows a sample from our prior. **C** Boxplots summarise each participant’s mean log EMG signal in each condition. The shaded region illustrates the 95% HPDI (highest posterior density interval) from an asymptotic regression model that ignores drug group information. Note: there are a number of outlier points that fall outside of this figure’s axis in both directions, and were omitted for clarity. CS, corrugator supercilii muscle; ZM, zygomaticus major muscle

The statistical model was fit using non-linear Bayesian regression with the *brms* package (v 2.18.1) for R (v 4.2.0) and Stan (v 2.26.13). We used normal (0, 0.2) and normal (− 1, 1) priors for estimating the fixed effect of *a* and* c*, respectively. See Fig. 2B for an illustration of these priors. The variances of all random effects were given a half-normal (0, 0.1) prior, while the residual variance used half-normal (0, 0.5). The model was run with 4 chains and 5000 iterations per chain. This resulted in a model with well-mixed chains with all R-hat statistics less than 1.01.

As had been found with frequentist statistics, a robust facial mimicry response was present for both emotions, irrespective of drug group. Thus, AngryToHappy videos elicited contraction of the ZM and relaxation of the CS (Fig. 2C, left). The reverse pattern was found for HappyToAngry videos (Fig. 2C, right).

**Fig. 3:**
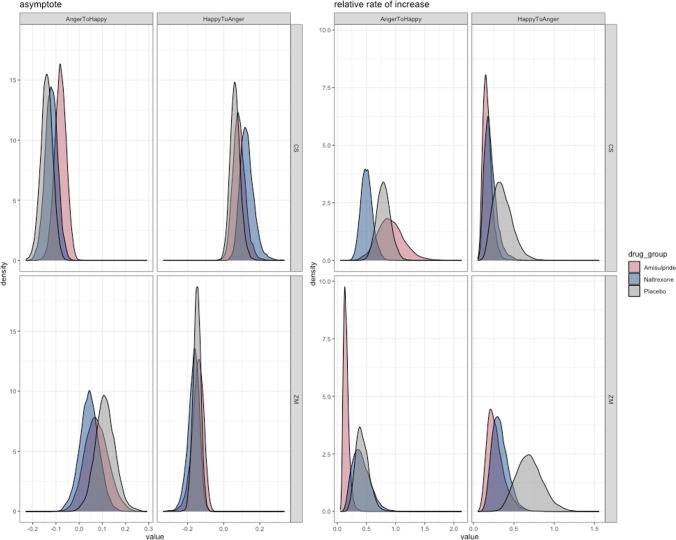
Posterior estimates for the fixed effects in our model. Remember that in our model rate of increase can only be positive, thus a greater rate for the ZM to HappyToAngry in the placebo group (lower right quadrant) should be interpreted as a greater relaxation of the muscle (towards the negative asymptote)

However, as can be seen from the model’s posterior distributions (Fig. 3), there is no clear effect of either amisulpride or naltrexone when compared to the placebo group.

To further explore the extent to which this is a true null result, we fitted a series of nested models. These simplified versions of the full model dropped the effect of drug group from either the asymptote, the rate of increase, or both variables. Model comparison was then carried out using Watanabe–Akaike information criterion (WAIC) and approximate leave-one-out (LOO) cross-validation, to obtain weights for each model. This procedure (see Table 2) revealed that the simpler model without drug effects received 99% of the model weight and should therefore be preferred over the more complex models including drug effects on either the asymptote, the rate of increase, or both (who all achieved less than 0.15% model weights). This provides strong evidence for the absence of a drug effect — although such effects might be found in future studies using measures of facial mimicry that result in stronger (and/or less variable) effects in the placebo condition.

**Table 2:** Model comparison metrics for nested models

Model	WAIC	LOO
Full	0.002	0.001
Drug effects asymptote only	0.013	0.127
Drug effects rate only	< 0.001	< 0.001
No effect of drug	0.995	0.985

*WAIC*, Watanabe–Akaike information criterion; *LOO*, approximate leave-one-out.

## Discussion

In order to shed light on the nature of facial mimicry, we tested the effects of amisulpride, a D2/D3 dopamine receptor antagonist, and naltrexone, a non-selective opioid receptor antagonist, on facial mimicry of happiness and anger. We found clear facial mimicry in all three participant groups, using a tried and tested task and a dynamic stimulus set. Thus, videos starting with decreasing anger and gradually changing into increasing happiness elicited greater contraction of the ZM muscle and relaxation of the CS muscle. In contrast, videos featuring the opposite change in expression, i.e. starting with decreasing happiness and gradually containing more anger, elicited ZM relaxation and CS activation. These changes in facial muscle activity, reflecting mimicry of the second emotion depicted in each video, were expected based on previous publications that utilised the same stimuli and paradigm (Korb et al. 2016a, b). However, neither drug significantly modulated the intensity of facial mimicry, as shown with both frequentist and Bayesian hierarchical models. In addition, response times — reflecting when participants perceived the change between emotions — were also not impacted by either drug. These null findings suggest that facial mimicry is not primarily driven by responses to rewards, as it remained unaffected by pharmacological modulation of both the ‘wanting’ and the ‘liking’ systems, respectively based on dopamine’s and opioids’ action. This stands in contrast to reports in the literature showing a modulation of facial mimicry by context and associated rewards.

Several non-pharmacological studies have indeed shown that facial mimicry is modulated by the reward value of the face stimuli encountered. For example, conditioning participants to associate particular face identities with winning money, compared to losing money, resulted in greater smile mimicry when these same faces were later shown dynamically changing from a neutral to a happy expression (Korb et al. 2019; Sims et al. 2014, 2012). Similarly, in an economic bargaining game, smile mimicry is suppressed in response to players who made unfair offers, resulting in participants losing money (Hofman et al. 2012). In addition, greater facial mimicry is often found when seeing emotions expressed by in-group members, which are more likely to be associated with rewards, compared to out-group members (Blocker and McIntosh 2016; de Klerk et al. 2019; van der Schalk et al. 2011; but see Sachisthal et al. 2016). These findings suggest that facial mimicry should be modulated by rewards, but the here-reported absence of a facial mimicry modulation by drugs targeting the dopaminergic and opioidergic systems, both playing a major role in the anticipation of and the hedonic responses to rewards, suggests that rewards should not be considered the primary driver of facial mimicry responses. Moreover, faces of in-group members can be both rewarding and self-relevant, and we recently showed that self-relevance can supersede reward value, when it comes to the modulation of facial mimicry (Forbes et al. 2021). In summary, although faces can be perceived as rewarding stimuli, especially when they display emotional expressions of positive valence, such as smiles, facial mimicry is not, based on current evidence, triggered primarily by reward seeking or reward consumption. As such, facial mimicry differs from the facial ‘liking’ responses to sucrose described in human infants, rats, and apes (Berridge and Kringelbach 2015) and from the more nuanced facial responses to food rewards (and to a lesser degree also touch) recently reported in adult humans (Korb et al. 2020a, b).

Dopamine and endogenous opioids have been established as the lead chemicals involved in the anticipation of and response to both social and non-social rewards (Berridge and Kringelbach 2015; Massaccesi et al. 2022), and previous research has shown that these neurotransmitter systems are reliably modulated by the here-used drugs and doses (Racagni et al. 2004; Trøstheim et al. 2023; Weber et al. 2016). Pharmacological challenges of the opioidergic system have however resulted in mixed effects on facial mimicry, i.e. reduced frowning (Meier et al. 2016) or no change in facial mimicry (Wardle et al. 2016) after administration of the opioid-receptor antagonist naltrexone, and reduced mimicry of fear (but not happiness nor anger) after administration of the mu-opioid receptor agonist morphine (Massaccesi et al. 2022). Measures of facial mimicry after a targeted modulation of the dopamine system are lacking, but greater corrugator relaxation in response to happy faces was found after administration of MDMA (Wardle and de Wit 2014), which mainly acts on the serotonin and noradrenaline system, but also influences the dopamine system.

Instead, we were unable to find a significant reduction of smile (as well as anger) mimicry after inhibition of D2/D3 dopamine and opioid receptors after administration of, respectively, 400 mg of amisulpride and 50 mg of naltrexone. This was the first measurement of facial mimicry after a targeted pharmacological challenge of the dopamine system in healthy participants, and the first direct comparison of the effects of dopaminergic and opioidergic antagonists on facial mimicry. Our null finding speaks against the (‘contextual’, or ‘mimicry as social regulator’) view that facial mimicry reflects a response that follows emotion understanding (Hess and Fischer 2022, 2014), given that at least happy faces should have been perceived as rewarding. Alternatively, our participants did not find the happy faces presented as rewarding, maybe because they were removed of any meaningful context or because they were preceded by angry faces. In that case, the clear presence of smile mimicry may be seen as further indication that the nature of facial mimicry is not a reward response — or at least not on every occasion. Unfortunately, we did not measure how rewarding (or self-relevant) each face was perceived, a limitation that future studies should try to overcome. Several other points could also be improved upon. First, a within-subjects design, in which every participant receives all drugs in counterbalanced order, might be preferable as it provides greater statistical power. Second, participants’ neurotransmitter baseline levels should be taken into account, as, for example, the effects of drugs affecting the dopamine system were previously shown to depend on baseline dopamine serum levels (Schuster et al. 2022). In this sample, dopamine serum levels were not taken into account, as they had previously been shown to play a minor role (Mikus et al. 2022), and we unfortunately did not dispose of measures of baseline opioid levels. Third, given that drug effects and especially the effect of dopaminergic drugs can be modulated by menstrual cycle, future studies should assess the menstrual cycle phase of female participants.

In conclusion, facial mimicry amplitude of happy and angry faces was not significantly modulated, neither by 400 mg of the D2/D3 dopamine receptor antagonist amisulpride, nor by 50 mg of the non-selective opioid receptor antagonist naltrexone. This null finding suggests that spontaneous facial mimicry is not primarily conducible to a reward/punishment response and does not support the claim that facial mimicry is an affiliative or strategic response to an already-perceived and recognised emotional expression. It is, however, compatible with the idea, advanced by theories of embodied cognition, that facial mimicry is an automatic motor copy, which can be, but is not necessarily, modulated by context and other factors. More research is however needed to conclusively establish the nature of facial mimicry, as it should be acknowledged that it is notoriously challenging to interpret null findings for novel effects.

## Supplementary Information

Below is the link to the electronic supplementary material.

Supplementary file1 (DOCX 364 KB)

## Data Availability

All the data and analysis scripts that support the findings are available in the OSF repository (https://osf.io/uht37).
