# Supplementary material for: Facial mimicry is not modulated by dopamine D2/3 and opioid receptor antagonism
Source: Psychopharmacology (Berl). 2023 Jul 21;240(10):2081–91. doi: 10.1007/s00213-023-06426-3 (PMC10506945; doi:10.1007/s00213-023-06426-3)
Supplement: Supplementary file 1 — Supplementary file1 (DOCX 364 KB) [file 213_2023_6426_MOESM1_ESM.docx]

Supplementary Materials


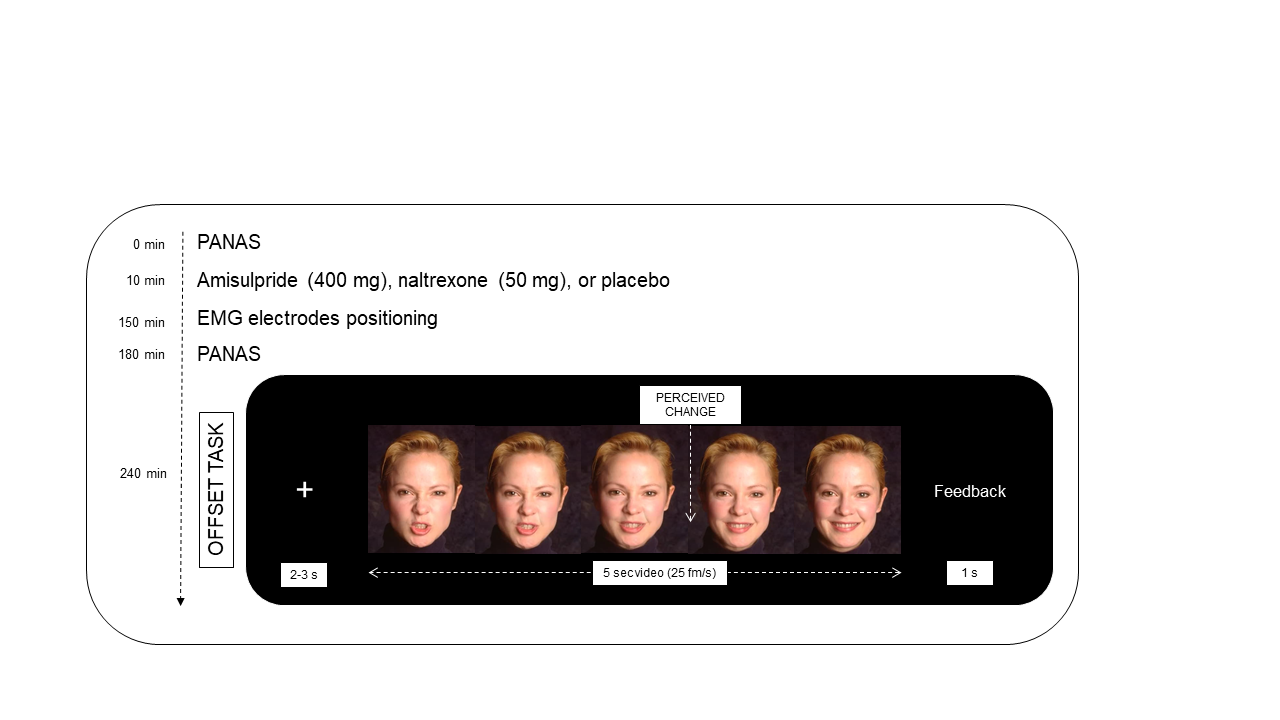


**Figure S1**. Experimental procedure, and example of an AngryToHappy trial in the offset task


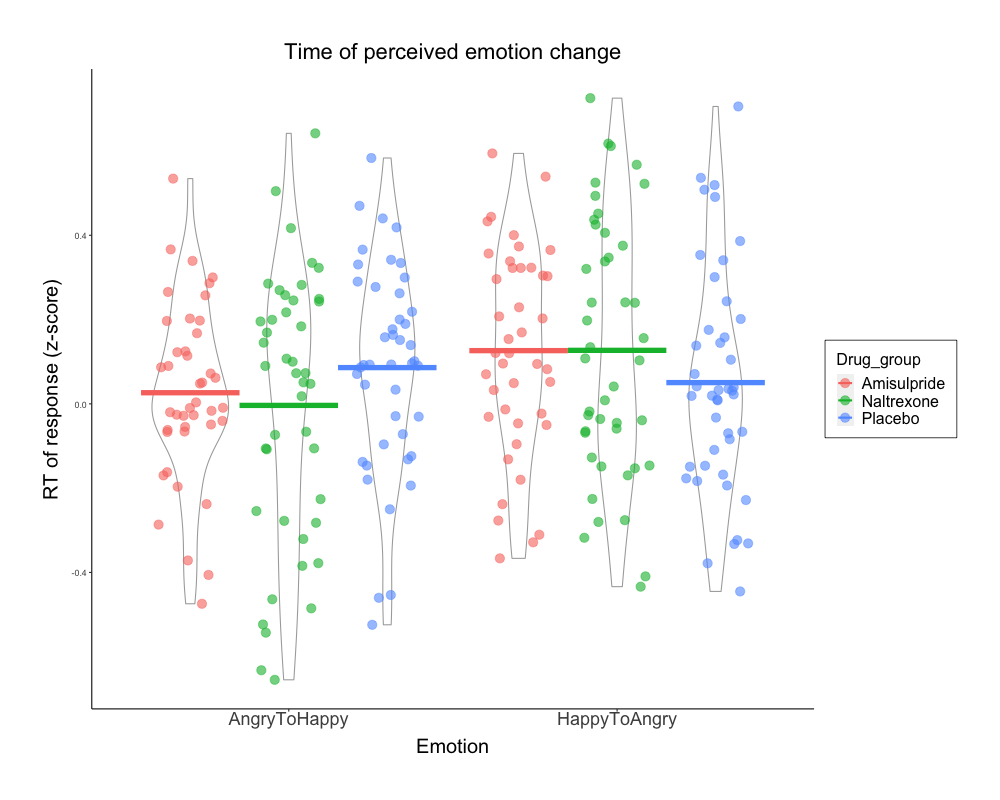


**Figure S2**. No drug effects were found on response times, for neither emotion. The horizontal lines represent group means. Individual dots show subject means.
